# Supplementary material for: Clinical presentation and management outcomes of pediatric lung abscess: A retrospective cohort study
Source: PLoS One. 2026 Mar 26;21(3):e0345174. doi: 10.1371/journal.pone.0345174 (PMC13020819; doi:10.1371/journal.pone.0345174)
Supplement: Attachment — Submitted filename: RESPONSE TO REVIEWER.docx [file pone.0345174.s002.docx]

**RESPONSE TO REVIEWER #1**

We thank the reviewer for the careful evaluation of our manuscript and for the constructive suggestions. We have revised the manuscript to clarify patient selection criteria, microbiologic evaluation, antimicrobial decision-making, and tuberculosis exclusion methods.

**1. Was immunosuppression/HIV excluded? Any children with recurrent infections?**

**Response:**
We appreciate this important point. Patients with known immunodeficiency disorders, including HIV infection, primary immunodeficiency, malignancy, or chronic immunosuppressive therapy, were excluded from the study. Additionally, none of the included patients had documented histories suggestive of recurrent severe infections or underlying immune compromise. This clarification has now been added to the Methods section.

**Manuscript Modification: Method/Study Population**

“Patients with known immunodeficiency disorders (including HIV infection, primary immunodeficiency, malignancy, or chronic immunosuppressive therapy) were excluded from the study. None of the included patients had documented histories of recurrent severe infections suggestive of underlying immune dysfunction.”

**2. Why were some cases started on Meropenem without microbiologic confirmation of MDR bacteria?**

**Response:**
We thank the reviewer for this important comment. In our institution, all patients with pediatric lung abscess were managed according to a standardized empiric protocol for severe necrotizing pulmonary infections. Initial therapy consisted of vancomycin combined with either a third-generation cephalosporin (ceftriaxone) or a carbapenem (meropenem), depending on clinical severity and extent of radiologic involvement.

Meropenem was selected in cases presenting with extensive necrosis, systemic toxicity, or concern for resistant Gram-negative pathogens, rather than based on confirmed multidrug-resistant organisms. Given the high rate of culture negativity in our cohort, antibiotic selection was guided by clinical severity and institutional protocols rather than microbiologic confirmation.

**Manuscript Modification: Discussion**

“This case series describes 23 pediatric lung abscess cases in a resource-limited setting, highlighting that most were primary abscesses in previously healthy children with prolonged symptoms before admission and variable severity. Despite limited resources, most patients responded well to IV combination antibiotics (aminoglycosides and beta-lactamase inhibitors) with step-down to oral therapy. Invasive interventions (surgery, bronchoscopy, ICU) were rarely needed, and most had full radiological resolutions.

These findings provide practical insights into the real-world management of pediatric lung abscess in similar healthcare settings and underscore the importance of timely diagnosis, structured antimicrobial therapy, and careful clinical monitoring. The use of broad-spectrum agents in selected severe cases reflects the pragmatic approach often necessary in necrotizing pulmonary infections, where delayed or inadequate treatment may increase morbidity. Nevertheless, these observations should be interpreted cautiously given the small sample size and retrospective design.”

**3. Why were there no microbiologic complementary exams (BAL or intraoperative samples)?**

**Response:**
We thank the reviewer for raising this important point. Bronchoalveolar lavage (BAL) was not routinely performed due to its invasive nature and because most patients were clinically stable and responded to medical therapy. Only one patient in the cohort required surgical intervention; in that case, intraoperative sampling was obtained for microbiological analysis. However, microbiologic yield remained low, likely influenced by prior antibiotic exposure. These clarifications have been incorporated into the Methods section.

**Manuscript Modification: Methods:**

“Bronchoalveolar lavage was not routinely performed due to its invasive nature and was reserved for selected severe or non-responding cases. In patients requiring surgical drainage, intraoperative samples were obtained for microbiological analysis when feasible.”

**4. TB exclusion – PPD is for latent TB; was direct exam/culture done?**

**Response:**

We thank the reviewer for this important clarification. Tuberculosis was excluded based on clinical assessment, radiological findings, and microbiologic testing when clinically indicated. A PPD test was performed in 30.4% (n=7) and TB PCR (GeneXpert) in 8.7% (n=2) of patients who had clinical or radiologic features warranting evaluation. In these cases, results were negative. No patient had clinical, radiologic, or microbiologic evidence suggestive of active tuberculosis. We have clarified this in the revised manuscript.

**Manuscript Modification: Result/ Patient Demographics and Baseline Characteristics:**

“Tuberculosis was considered in the differential diagnosis and excluded based on clinical evaluation, radiologic assessment, and microbiologic testing when clinically indicated. A tuberculin skin test (PPD) was performed in 30.4% (n=7) of patients, and TB PCR (GeneXpert) was performed in 8.7% (n=2) of cases with clinical suspicion. All results were negative. No patient demonstrated clinical, radiologic, or microbiologic evidence of active tuberculosis.”

**Minor Revisions**

- **Replace “Tazocin”**

Done

- **Tables should be simplified**

We have revised and simplified the tables to improve clarity and readability.
